# Quality of Life and Cognitive Function Evaluations and Interventions for Patients with Brain Metastases in the Radiation Oncology Clinic

**DOI:** 10.3390/cancers14174301

**Published:** 2022-09-01

**Authors:** Jennifer K. Matsui, Haley K. Perlow, Cyril Baiyee, Alex R. Ritter, Mark V. Mishra, Joseph A. Bovi, Vinai Gondi, Paul D. Brown, Ashlee R. Loughan, Heather E. Leeper, Erica Dawson, Joshua D. Palmer

**Affiliations:** 1 College of Medicine, The Ohio State University, Columbus, OH 43210, USA; 2 Department of Radiation Oncology, The Ohio State University Wexner Medical Center, Columbus, OH 43210, USA; 3 Department of Radiation Oncology, University of Maryland, Baltimore, MD 20742, USA; 4 Department of Radiation Oncology, Medical College of Wisconsin, Milwaukee, WI 53226, USA; 5 Department of Radiation Oncology, Northwestern Medicine Cancer Center and Proton Center, Warrenville, IL 60555, USA; 6 Department of Radiation Oncology, Mayo Clinic, Rochester, MN 55905, USA; 7 Department of Neurology, Division of Neuro-Oncology, Virginia Commonwealth University, Richmond, VA 23284, USA; 8 Neuro-Oncology Branch, National Cancer Institute, National Institutes of Health, Bethesda, MD 20814, USA; 9 Department of Neurology, The Ohio State University Wexner Medical Center, Columbus, OH 43210, USA

**Keywords:** brain metastases, radiotherapy, quality of life, cognition, cognitive function

## Abstract

**Simple Summary:**

Brain metastases (BMs) are the most common brain malignancy and are projected to increase in incidence over the coming decades. Historically, brain metastasis studies have focused on improving survival outcomes, but recently, the importance of evaluating health-related quality of life (HRQOL) and cognitive function has gained recognition. Although there is a myriad of validated HRQOL and cognitive assessments available in the radiation oncology clinic, there is an urgent need to identify tools tailored to patients with BMs and to adopt a uniform set of tests that measure HRQOL and cognition. This review presents various assessments for measuring HRQOL and cognitive function, current recommendations to improve standardization, and treatments known to preserve HRQOL and cognitive function.

**Abstract:**

Brain metastases (BMs) account for a disproportionately high percentage of cancer morbidity and mortality. Historically, studies have focused on improving survival outcomes, and recent radiation oncology clinical trials have incorporated HRQOL and cognitive assessments. We are now equipped with a battery of assessments in the radiation oncology clinic, but there is a lack of consensus regarding how to incorporate them in modern clinical practice. Herein, we present validated assessments for BM patients, current recommendations for future clinical studies, and treatment advances that have improved HRQOL and cognitive outcomes for BM patients.

## 1. Introduction

Brain metastases (BMs) occur in approximately 20% of all cancer patients and are the most common brain neoplasm [1]. Survival outcomes are improving for Stage IV cancers, which are unfortunately associated with higher BM incidence [2]. The life expectancy of BM patients has increased due to advances in radiation treatment (RT) and systemic therapy [3]; therefore, maintaining health-related quality of life (HRQOL) and cognitive function during and after treatment are imperative. Many clinical trials have included HRQOL and cognitive function assessments, but there is not yet a standardized clinical approach to assessing these patients in modern clinical practice. There is a pressing need to (1) identify tools tailored to BM patients and (2) adopt a uniform set of tests that measure HRQOL and cognition that will facilitate the design of high-quality studies that can improve the lives of BM patients.

In this review, we highlight validated tools available for measuring HRQOL and cognitive function, current recommendations to improve standardization and clinical application, and advances in RT and systemic therapy that have improved HRQOL and cognitive function.

## 2. Treatment of Brain Metastases

### 2.1. Radiation

Historically, whole-brain radiation therapy (WBRT) was the primary radiation treatment for BMs. WBRT can treat local and distant intracranial disease through radiating the entire brain, including the leptomeninges [4]. Patchell et al. demonstrated that the addition of surgery to WBRT increases median overall survival (OS) and functional independence [5]. Subsequently, Patchell et al. reported that WBRT in the post-operative setting reduces recurrence and neurologic death, but did not have a significant impact on OS [6].

The QUARTZ trial was a non-inferiority trial comparing outcomes in NSCLC patients with BMs unsuitable for surgical resection or SRT, many of whom had poor performance status, who received either optimal supportive care (OSC) plus WBRT or OSC alone [7]. In this trial, patient-reported quality-adjusted life-year (QALY) (a measure of disease burden accounting for quality and quantity of life lived) was designated as the primary outcome, given the poor prognosis for their patient population. In their study, patients receiving WBRT were reported to have significantly more episodes of drowsiness, alopecia, nausea, and dry or itchy scalp. Although the younger patients (<60 yo) and patients with ≥5 BMs groups appeared to attain improved OS (patients <60 yo had a HR of 1.48 (CI 1.01–2.16) and patients with ≥5 BMs had a HR of 1.37 (CI 1.01–1.86)), there was not a significant difference detected in the entire cohort. Chang et al. found that while combining SRS and WBRT led to an improvement in PFS, there was a significant decline in learning and memory by 4 months compared to SRS alone [8].

Brown et al. also reported greater cognitive deterioration in patients receiving SRS and WBRT versus SRS alone (91.7% vs. 63.5%) 3 months post-RT [9]. Li et al. conducted a phase III randomized trial that also suggested cognitive function decline was improved with SRS alone at 4 months, despite again a demonstration of improved OS [10]. Therefore, the benefits of WBRT must be weighed against the risks of radiation-related toxicity (e.g., skin erythema, alopecia, fatigue), as well as chronic adverse effects such as dementia, confusion, and leukoencephalopathy [11].

In an effort to reduce radiation-induced cognitive deterioration, Brown et al. hypothesized that hippocampal-avoidance WBRT (HA-WBRT) with memantine may prevent neurocognitive toxicity. In NRG CC001, they found that hippocampal sparing significantly reduced the risk of cognitive failure, as evidenced by less executive function deterioration at 4 months and learning memory deterioration at 6 months. Further, patients receiving hippocampal sparing plus memantine reported less fatigue, difficulty remembering, and difficulty with speaking [12].

### 2.2. Surgery

Surgical intervention is important in the management of BMs. Early intervention can relieve symptomatic mass effect and establish a diagnosis for patients with no prior history of cancer [4]. Surgical candidates with a single lesion, good Karnofsky Performance Status (KPS), and a limited number of extracranial metastases have improved survival outcomes [5,13]. Gross total resection (GTR) is favored over subtotal resection with the caveat that an aggressive resection is not likely to result in further neurological deficit [14,15]. An en bloc resection (circumferential dissection along the brain–tumor interface) versus a piecemeal resection has been found to decrease the risk of local recurrence and leptomeningeal dissemination [14,16,17]. More recently, intraoperative neuronavigation, cortical mapping [18], and convection-enhanced delivery [19] are techniques used to improve the GTR rate and morbidity. Laser interstitial thermal therapy (LITT) is a procedure increasing in popularity that involves neurosurgical stereotactic placement of a probe that kills tumor tissue with heat [20,21]. In patients with BMs, avoiding functional neural tissue with this targeted procedure should theoretically lead to less neurocognitive decline compared to more invasive techniques [22]. A prospective trial reported patients that develop radiation necrosis or recurrence benefit from LITT [23].

### 2.3. Systemic Therapy

Although systemic therapy is used to treat patients with disseminated disease, the blood–brain barrier causes low rates of intracranial penetrance. Multiple studies have reported that chemotherapy does not improve OS in BM patients that received WBRT [24], although more recent studies have suggested certain cancer patient populations (e.g., NSCLC, breast, melanoma) may benefit [25]. Osimertinib, a tyrosine kinase inhibitor, has been approved for patients with metastatic non-small cell lung cancer, the leading cause of BMs [26]. Osimertinib has intracranial efficacy with acceptable toxicity [27]. Patients with HER2-positive intracranial metastatic breast cancer have also benefited from targeted agents such as tucatinib combination therapy [28]. Dabrafenib plus trametinib is another targeted therapy for patients with BRAFV600-mutant melanoma that has demonstrated intracranial response in patients with BMs [29]. A phase II study utilizing ipilimumab reported a modest response for melanoma patients with BMs, but is currently not recommended as standard treatment for larger or symptomatic BMs. Although these results are encouraging, not all patients with BMs are eligible for these targeted agents. There is an ongoing debate whether upfront systemic therapy is appropriate in lieu of established local therapies.

### 2.4. Symptom Control

Corticosteroids and antiepileptic agents are commonly used in BM patients for symptom control. Dexamethasone is a common corticosteroid used to control peritumoral edema and reduce elevated intracranial pressure (ICP) [30]. Asymptomatic patients typically do not receive corticosteroids [31,32], but patients with more severe symptoms due to increased ICP benefit from steroids [33]. The Quality of Life after Treatment for Brain Metastases (QUARTZ) trial compared optimal supportive care (including dexamethasone) plus WBRT to optimal supportive care alone in patients unsuitable for surgical resection or SRS [7]. This study found that WBRT provided little additional clinically significant QOL or OS benefit in this patient population. While dexamethasone may provide temporary symptomatic relief, the American Society of Clinical Oncology and the Society for Neuro-Oncology recommend corticosteroids should be tapered as rapidly as possible [34]. Ideally, steroids should be tapered and discontinued within 2 weeks after initiation to avoid chronic steroid use effects [31].

Seizures can occur in up to 25% of BM patients, but there is limited level 1 evidence regarding the role of antiepileptic agents [35]. Results from meta-analyses suggest that the use of antiepileptic drugs in patients without a seizure history provides no immediate or long-term benefit in BM patients [36].

## 3. Radiation-Induced Cognitive Decline: Mechanisms of Action

Radiation is used to treat many primary brain tumors, BMs, head/neck cancer, and leukemia/lymphoma involving the central nervous system. Months to years after radiation exposure, many patients experience deficits in memory, spatial relations, visual motor processing, quantitative skills, and attention [37,38].

Radiation-induced brain injury can either be acute, subacute (6 months post-RT), and/or chronic [39] and remains an incompletely understood, yet active area of research. A common presentation of acute RT injury is acute encephalopathy, which can result from a high dose per fraction (e.g., above 3 Gy per fraction) [40,41]. Subacute complications include somnolence syndrome, defined by a group of symptoms including extreme drowsiness, clumsiness, lethargy, and slow mental processing. These symptoms are concerning because they can be irreversible and lead to progressive dementia [42]. Although cognitive deterioration related to RT is multifactorial, a hypothesized large driving factor is the decrease in neurogenesis [43]. Neurogenesis occurs in critical regions of the brain such as the subgranular zone of the hippocampus and subventricular zone of the lateral ventricles [44].

Historically, radiation injury was thought to arise through two mechanisms: radiation-induced vascular injury and radiation-induced ablation of glial precursors. However, neither hypothesis explains why most patients with significant cognitive deterioration lack signs of overt vasculopathy or demyelination [45]. Another hypothesis proposes radiation may damage the hippocampal granule cell layer, which undergoes neurogenesis.

The hippocampus has been identified as a prominent brain structure responsible for consolidation and retrieval of newly learned information [46]. The hippocampus is located in the ventromedial area of the temporal lobes, lateral to the temporal horn of the lateral ventricle, and is composed of the dentate gyrus and the cornu ammonis [47]. Neural stem cells are located in the subependymal zone and subgranular zone of the dentate gyrus and are capable of self-renewal [48].

Preclinical studies demonstrated that neural stem cells are sensitive to ionizing radiation. In a young adult rat, a single dose of 5 or 30 Gy caused apoptosis of neural stem cells in the subependymal zone [49]. A subsequent study found a single 10 Gy dose, a clinically relevant dose in humans, led to apoptosis in the rats’ dentate gyrus [50]. Monje et al. demonstrated that the decrease in hippocampal neurogenesis is accompanied by alterations in the microenvironment with an increase of microglia [51]. Even a dose of 2 Gy to human neural stem cells was reported to lead to a decrease in cells undergoing neural differentiation [52].

Although late toxicity data post-WBRT are limited due to the short median survival time, clinicians have noted that bilateral and unilateral radiation injury of the hippocampus led to deficits in learning and memory formation [53]. One study that evaluated 1-year survivors of a single brain metastasis who received WBRT found no decrease in neurocognitive function nor an increase in leukoencephalopathy if the fraction size was <3 Gy [54]. Another study by Sheline et al. found that a WBRT fraction size of 2.5 Gy was associated with decreased risk of neurocognitive decline [39]. Although there is still a lack of consensus, these findings collectively suggest the utilization of low-dose per fraction with WBRT may be efficacious.

## 4. Measuring Quality of Life and Neurocognitive Function in the Radiation Oncology Clinic

Historically, improving QOL was secondary to the goals of improving survival outcomes in numerous clinical trials. Although there is still a focus on maximizing survival outcomes, clinicians are recognizing that QOL and cognitive preservation are essential [55]. Measuring QOL is challenging due to the subjectivity of physical and psychosocial factors [56]. Additionally, many patients with BMs experience neurocognitive dysfunction at the time of diagnosis; therefore, establishing an accurate premorbid baseline is often not feasible, yet striving for a pretreatment baseline is essential. Researchers and practitioners in modern clinical practices have sought to design neurocognitive tests that balance practicality (e.g., can be administered by staff who do not require neuropsychological expertise) and sensitivity [8,57,58]. Below, the most common tools for measuring cognition and quality of life are discussed, although this is not all-encompassing of all available testing options for patients. Moreover, cognitive assessments are a burgeoning area of focus internationally, where greater variability in educational attainment/language spoken make this a particular challenge and area of great interest in neuropsychology.

### 4.1. Patient-Reported Outcome Questionnaires

#### 4.1.1. EQ-5D

The EQ-5D is a series of QOL questionnaires that have been validated as a tool for cancer patients [59,60,61,62]. The questionnaires are brief and include items measuring mobility, self-care, completion of usual activities, pain/discomfort, and anxiety/depression. On the simplest form, items are simply marked as being present or absent, whereas on other forms, patients can indicate symptom severity. There is also an item measuring overall perceived health, rated on a scale of 0–100.

#### 4.1.2. Functional Assessment of Cancer Therapy-General

The Functional Assessment of Cancer Therapy-General is a 33-item questionnaire that was designed to measure QOL in cancer patients [63]. Symptom domains include physical, social/family, emotional, and functional well-being. Each item is rated on a scale from 0 to 4, and some items are reverse-scored.

#### 4.1.3. Functional Assessment of Cancer Therapy-Brain

A supplementary set of items, referred to as the FACT-Brain (FACT-Br), assesses neurological symptoms that can occur secondary to primary central nervous system tumors, such as cognitive and sensory complaints [64]. Although the tool was originally designed for patients with primary brain tumors, the FACT-Br demonstrated effectiveness for assessing patients with BM [65].

#### 4.1.4. EORTC Quality of Life Questionnaire

The EORTC QLQ-C30 is a questionnaire designed to assess the QOL of cancer patients [66,67]. This tool is a multi-dimensional HRQOL that is composed of six functional scales (e.g., ability to walk, wash self), three symptom scales (e.g., shortness of breath, trouble sleeping), and additional single-item scales. There is a variety of validated models for specific disease sites (e.g., cervical cancer, colorectal cancer), including one dedicated to brain cancer (EORTC QLQ-BN20). Numerous studies have utilized the QLQ-C30 alongside the QLQ-BN20 for BM patients [68,69]. Other studies have suggested that the QLQ-BN20 in conjunction with the QLQ-C15-PAL (Core 15 Palliative) is an effective way to measure QOL in BM patients with a lower question burden [70].

A summary of the patient-reported outcome questionnaires is outlined in Table 1.

### 4.2. Neurocognitive Tests

#### 4.2.1. Mini-Mental State Examination

The Mini-Mental State Examination (MMSE) is a popular tool for measuring neurocognitive outcomes. This exam consists of 30 question items and can be completed in under 10 min [74]. This test measures orientation to time and place, short-term memory recall, attention, working memory, language, and other basic neurocognitive skills. Although large differences in MMSE scores can reliably indicate clinically significant deterioration in cognitive function, it is often not a suitable tool for detecting changes in memory function, executive function, and psychomotor speed seen in patients with brain tumors due to limited sensitivity [75]. In fact, in patients with primary CNS tumors, the MMSE was no more sensitive to cognitive impairment than a coin toss [76]. Further, the RTOG 0214 demonstrated the limitations of the MMSE. A phase III randomized trial compared non-small cell lung cancer patients who either underwent observation or prophylactic cranial irradiation (PCI) [77]. While the Hopkins Verbal Learning Test (HVLT) indicated deterioration in memory, the MMSE demonstrated no between-group differences [78].

#### 4.2.2. The Montreal Cognitive Assessment

The Montreal Cognitive Assessment (MoCA) is another widely utilized cognitive test that assesses short-term memory recall, visuospatial abilities (clock drawing and copying a three-dimensional cube), executive function, attention, language, abstract reasoning, and orientation to time and place [79]. The MoCA was hypothesized to be an effective cognitive assessment tool for brain tumors for the following reasons: (1) the MoCA has greater sensitivity than the MMSE in capturing mild cognitive impairment; (2) the test is less than 10 min (potentially increasing compliance); (3) the test is more extensive than the MMSE (e.g., assesses attention, learning, and executive function at a greater depth) [80]. Olson et al. compared the MMSE and the MoCA and found that the MoCA had greater sensitivity and better correlation with self-reported quality of life measures (61.9% vs. 19.0%, *p* < 0.005) [81]. Although this test has greater sensitivity than the MMSE, it may not be an ideal screening tool for detecting small changes in cognitive function experienced by patients with brain tumors [82].

#### 4.2.3. Repeatable Battery for the Assessment of Neuropsychological Status

The Repeatable Battery for the Assessment of Neuropsychological Status (RBANS) consists of 12 subtests that assess immediate memory, visuospatial abilities, language, attention, and delayed memory [83]. The test takes approximately 30 min to administer and has two forms for serial testing. Historically, this test has been extensively used for patients with dementia, multiple sclerosis, Parkinson disease, and other neurological disorders [84]. Although there is currently limited studies utilizing the RBANS in neuro-oncology, some studies have found the RBANS to be an effective screening tool for patients with primary brain tumors [85].

#### 4.2.4. Trail Making Test

The Trail Making Test (TMT) is a timed neuropsychological test that assesses processing speed and attention shifting. There are two parts to the test. Part A tests visual scanning and sequencing; patients are asked to connect numbers 1–25 in ascending order, which are scattered on a piece of paper. Part B tests attention shifting [86]; patients are asked to connect numbers and letters in alternating sequencing in ascending and alphabetical order, which are also scattered on a piece of paper. The test is scored based on completion time (including time necessary to correct errors), balancing speed and accuracy. Although this test was originally designed to detect cognitive impairment in dementia patients, it is extensively used across patient populations, including BM patients [87].

#### 4.2.5. Hopkins Verbal Learning Test-Revised

The Hopkins Verbal Learning Test-Revised (HVLT-R) includes three learning trials of 12 orally presented words, a 25-min delayed recall trial, and a recognition trial during which patients are asked to identify words with a “yes” or non-target words with a “no”. Over the past two decades, the HVLT-R has demonstrated reliability and validity across clinical populations [88,89]. Numerous RT BM trials have utilized this test to assess the effects of RT on neurocognitive function [8,78,90].

#### 4.2.6. Controlled Oral Word Association Test

The Controlled Oral Word Association Test (COWAT) is a measure of verbal fluency. During the phonemic fluency trials, patients are given three 1 min opportunities to state as many words as possible that begin with a specified letter [91]. The test is scored based on the summation of different words produced for all three letter trials. Error patterns (e.g., repetition of a word) are also noted [92]. The resulting scores are useful in evaluating patients with stroke, traumatic brain injury, and dementia, but have also demonstrated sensitivity in patients with BMs [93].

A summary of the neurocognitive tests are outlined in Table 2.

## 5. Current Recommendations for Assessing HRQOL and Neurocognition

Neuropsychological evaluations are considered the “gold standard” for evaluating cognitive function, especially for clinical purposes [97]. Neuropsychological evaluations are particularly powerful given the flexibility of tailoring tests administered to assess specific cognitive functions combined with patient-specific treatment recommendations rendered based on each individual’s neuropsychological profile [98]. The primary drawback of these assessments is accessibility, particularly for research purposes [99]. In the context of research, specifically, there has been an effort to establish standardized neurocognitive tests that can be completed in a reasonable amount of time with adequate sensitivity by appropriately trained research staff.

The current recommendations for evaluating HRQOL and neurocognitive function in patients with BMs stem from previous recommendations for patients with non-CNS and glioma tumors; these recommendations are briefly discussed below to provide greater context. The recommendations for patients with BMs are outlined at the end of this section.

### 5.1. Patients with Non-CNS Tumors

The International Cognition and Cancer Task Force (ICCTF) has presented cognitive test recommendations for patients with non-CNS tumors; these tests include the HVLT-R, Trail Making Test (TMT), and Controlled Oral Word Association Test (COWAT) for patients with non-CNS tumors [100]. When choosing specific tests, the ICCTF focused on measuring learning and memory; processing speed; and executive function. The goal was assessing cognitive domains that would be affected by chemotherapy (e.g., frontal subcortical circuitry). Additionally, the ICCTF selected tests with adequate sensitivity that require little overall time to administer.

### 5.2. Patients with a Glioma

The Response Assessment in Neuro-oncology (RANO) criteria suggest that the assessment of clinical benefit or deterioration in low-grade gliomas should include cognitive function tests and HRQOL (e.g., measuring symptom burden) [101]. To assess cognitive dysfunction, they recommend using the MMSE to stratify patients at baseline. Additionally, the RANO suggests utilizing a series of more sensitive neurocognitive tests (HVLT-R, TMT (Parts A and B), COWAT) at baseline and at longitudinal follow-up time points. These tests are designed to measure memory, executive function, and processing speed in a reasonable timeframe (20–30 min). Regarding the measurements of HRQOL, the EORTC QLQ-C30 with QLQBN-20, EQ-5D-3L or -5L, or FACT-BR have each demonstrated robust psychometric properties and can be completed within 5-20 min [102,103]. The HRQOL questionnaires should be administered before treatment initiation, at regular intervals during and after treatment, and continued in the event of tumor progression [100,101,104].

### 5.3. Patients with Brain Metastases

For the patients with BMs, the RANO-BM and ICCTF recommend using the HVLT-R, TMT (Parts A and B), and COWAT to assess neurocognitive function [105]. These tests should be administered at various time points to distinguish acute versus long-term treatment toxicity. Although neurocognitive tests do not always correlate with QOL, there is evidence that neurocognitive decline is associated with a reduction in HRQOL and ADLs [75]. To assess HRQOL in patients with BMs, the RANO recommends using the following validated tools: the EORTC QLQ C30 and QLQ BN-20, FACT-Br, or EQ-5D-3L or -5L. The RANO recommends clinical trials include endpoints of QOL and neurocognitive function in later-phase studies.

## 6. Challenges Measuring and Interpreting Quality of Life and Cognitive Outcomes

From the early 2000s, neurocognitive tests post-RT have been included in randomized control trials, but the actual impact of RT has been difficult to define due to the lack of standardized measurements. There is also a lack of standardized HRQOL tools specifically designed for patients with BM that measure important factors (e.g., well-being, pain, mood).

Existing neurocognitive evaluations and QOL measurements available for cancer patients are typically lengthy, which may limit feasibility by increasing participant burden in clinical trials. One study found the compliance rate was 56% at 6 months for cancer patients completing self-reported QOL examinations [106]. Clinicians have recognized the importance of using succinct tools to lower question burden for patients. Walker et al. found the largest cause of missing data was administrative failure; they recommended that studies monitoring QOL find avenues to minimize sources of missing data and record reasons for non-compliance. Bae et al. evaluated patient factors associated with missing data using a variety of brain cancer trials and found institutional error and request to not be contacted were frequent causes for missing data, but a majority of cases were unspecified [94].

Verhaak et al. conducted a systematic review of HRQOL outcomes for BM patients who received SRS [107]. Although some studies reported stable HRQOL scores at the group level, individual changes have been challenging to deduce given that test scores can remain constant in the event of improvement in some symptoms and declines in others [108]. Furthermore, different questionnaires were utilized across studies (e.g., EQ-5D, FACT-Br), leading to incongruent results; studies that used EQ-5D reported a decline in physical HRQOL [109,110,111], whereas studies using FACT-Br reported stable scores over time [112,113,114]. This lack of standardization prevents the pooling of study results for meta-analyses.

The setting where the questionnaire is completed may also affect the results. In some studies, follow-up questionnaires are sent via mail [112]. Although there is a possibility that completing the forms at home could induce less stress or anxiety than in the hospital setting, there is also a possibility that the patients will be influenced by others or not complete the test correctly [107]. Patients may also lose the questionnaires and/or be unmotivated to complete the questionnaires once returning home.

Furthermore, interpreting HRQOL data from patients can be complicated by a range of other factors including the effects of non-radiation treatment (e.g., chemotherapy, immunotherapy, surgery), additional medication (e.g., steroids, anti-depressants), and disease progression.

## 7. Strategies for Improving Cognition and Quality of Life in Brain Metastases Patients

### 7.1. Omitting Whole-Brain Radiation Therapy

Although WBRT significantly improves tumor control after SRS, the utilization of WBRT remains controversial due to its association with cognitive decline and decreased QOL [8]. Brown et al. addressed this controversy by comparing cognitive deterioration at 3 months after SRS plus WBRT versus SRS alone [9]. In this randomized trial, patients with 1 to 3 BMs treated with SRS alone experienced less cognitive deterioration, better QOL, and no difference in OS. These findings suggest patients with three or fewer BM may benefit from the omission of WBRT.

### 7.2. Utilizing Stereotactic Radiosurgery

Compared to WBRT, SRS is highly precise with a sharp dose gradient and has the ability to spare healthy brain tissue, reducing the risk of long-term toxicity [115,116]. Verhaak et al. conducted a systematic review of HRQOL in BM patients who received SRS [107]. Of the nine reviewed studies, four reported stable HRQOL up to 12 months following RT [58,112,113,114] and three studies found a decline in overall HRQOL. Several of these studies noted a decline in HRQOL after disease progression. Serizawa et al. conducted a multivariate analysis of patients undergoing SRS (up to 10 metastases) and found that involvement of the temporal lobe, parietal lobe, and brainstem resulted in QOL decline [117].

Bunevicius et al. conducted a prospective study evaluating QOL in BM patients who underwent SRS [118]. QOL was measured using the EQ-5D index score, which found no statistically significant change between the first and last post-SRS visit (first post-SRS visit was at median of 2.59 months; last post-SRS visit was a median of 14.72 months). Predictors of post-SRS QOL deterioration included a higher recursive partitioning analysis (RPA) class, upfront WBRT, and greater intracranial disease burden.

SRS is an excellent option for suitable BM patients, which can result in cognitive preservation with equivalent OS [119,120]. With the increasing availability of SRS and the concerns regarding WBRT-related cognitive decline, more radiation oncologists are treating BM patients with SRS [2,11]. Historically, SRS alone was used to treat 1 to 3 lesions, but prospective trials suggest treating 5 to 10 lesions is also safe and effective [116].

### 7.3. Hippocampal-Avoidance Whole-Brain Radiation Therapy

WBRT has been associated with a decline in memory and patient-reported QOL [121]. Following reports of memory decline in WBRT patients, hippocampal avoidance using intensity-modulated radiation therapy (IMRT) has been explored to reduce the radiation dose to the hippocampus [122,123]. The hippocampus is thought to contain cells necessary for neurogenesis that are exquisitely radiosensitive [51].

The RTOG 0933 demonstrated superior cognitive preservation with hippocampal avoidance [124]. The RTOG 0933 reported patients receiving HA-WBRT versus standard WBRT had a mean relative decline of 7% and 30% at 4 months, respectively (*p* < 0.001). Subsequently, the NRG-CC001 trial compared WBRT with HA-WBRT with both arms receiving memantine post-RT [125]. Standardized neurocognitive function (NCF) tests were performed at baseline, and the primary endpoint was time to NCF failure. The HVLT-R, TMT, and COWAT were used to detect NCF failure. Neither treatment arm had a significant difference in baseline NCF, OS, or intracranial progression-free survival, but the time to NCF failure was significantly longer in the HA-WBRT with memantine arm.

Hippocampal sparing was also explored in patients undergoing prophylactic cranial irradiation (PCI) by Redmond et al. [126]. Patients with small cell lung cancer (SCLC) were prospectively evaluated for cognitive function and intracranial failure patterns following hippocampal-sparing PCI and were found to have no significant decline in performance on the HVLT-R, COWAT, and TMT between baseline and 12 months. More recently, the PREMER phase III study evaluated the incidence of BM within the hippocampal avoidance zone in SCLC patients receiving hippocampal-avoidance-PCI [127]. In this study, delayed free recall was assessed using the Free and Cued Selective Reminding Test at 3 months. The authors found that sparing the hippocampus led to less cognitive decline with no differences in intracranial relapse, OS, and QOL compared to standard PCI. Finally, the NCT01780675 phase III trial reported that patients with SCLC receiving HA-PCI did not have an increase in BMs at 2 years or a lower probability of cognitive decline compared to conventional PCI [128]. The NRG-CC003 is an ongoing phase II/III trial for patients with SCLC evaluating whether HA-PCI (1) results in non-inferior intracranial relapse rates and (2) reduces cognitive deterioration at 6 months compared to conventional PCI.

### 7.4. Fractionated Treatment

Accelerated hyperfractionation (AH) WBRT has been explored as an alternative to accelerated fractionation (AF) WBRT for BM treatment. Theoretically, administering multiple daily fractions should decrease the tumor cell repopulation during RT without an increase in late tissue toxicity [129]. To assess the effects of altered fractionation, the RTOG 91-04 compared AH-WBRT (54.4 Gy in 1.6 Gy BID fractions) with AF-WBRT (30 Gy in 3 Gy fractions) in patients with unresected BM [130]. Regine et al. reported there was not a significant difference in neurocognitive outcomes (measured by the MMSE), but acknowledged the limitations of using a single global screening tool to measure neurocognition. ALLIANCE A071801 is an ongoing trial comparing QOL in single versus fractionated SRS to assess whether fractionation may decrease local failure (LF). Many validated tests (e.g., FACT-Br, Linear Analog Self-Assessment (LASA) overall QOL) are used in this study, which may detect differences in QOL outcomes caused by a hypothesized increased rate of LF [131].

### 7.5. Neuroprotective Agents

Memantine is an *N*-methyl-D-aspartate (NMDA) receptor antagonist that has been used as a neuroprotective drug for dementia [132,133,134]. Researchers became interested in the utility of memantine as a neuroprotective agent for brain irradiation after promising preclinical findings [135]. The RTOG 0614 found memantine may delay time to cognitive decline and reduce the rate of decline in memory and executive function in patients undergoing WBRT [136]. The primary endpoint did not reach statistical significance, likely due to significant patient loss, but the benefits of memantine have still led to widespread adoption in the radiation oncology clinic. Investigators randomized patients to receive memantine or placebo in addition to WBRT. Memantine was given daily with WBRT and afterwards for a total of 24 weeks. The primary endpoint was preservation of cognitive function, which was assessed with the HVLT-R at 24 weeks. Patients who received memantine experienced significantly longer time to cognitive decline (HR 0.78, 95% CI 0.62–0.99, *p* = 0.01). The memantine patients also had superior COWAT performance at 16 weeks (−0.05 versus −0.42, *p* = 0.038) and for the Trail Making Test Part A at 24 weeks (0.075 versus −0.37, *p* = 0.014). Although reports show that memantine attenuates radiation-induced cognitive decline, there is room for improvement (at 24 weeks post-RT, cognitive preservation was 31% with memantine and 20% with placebo). Attention-enhancing medications such as methylphenidate and modafinil have failed to improve symptoms or QOL [137,138].

### 7.6. Cognitive Rehabilitation Training

Cognitive rehabilitation therapy provides exercises that are designed to improve various domains of cognition: attention, memory, language, and executive function [139]. The techniques that are utilized are retraining (repeating targeted exercises) and compensation (patients are encouraged to create goals that increase functionality) [140]. Studies have found that patients with brain-tumor-related epilepsy with cognitive deficits benefited from cognitive rehabilitation training [141]. Notably, Maschio et al. found short- and long-term verbal memory significantly improved with training. Neurocognitive rehabilitation has shown to be effective for patients recovering from chemotherapy, bone marrow transplant, or as a part of cancer survivorship [142,143,144,145]. Historically, rehabilitation might not have been presented to many brain metastases patients given the quick decline following diagnosis and short life expectancy. However, with new radiographic and system therapies demonstrating promising life extension, this is an important future area of inquiry. There is one known study prospectively evaluating neuropsychologic rehabilitation for patients receiving radiation for brain metastases (NCT05503251).

## 8. Conclusions

BMs are currently the most common brain tumor in adults and are projected to increase in incidence as systemic therapies and other treatment modalities improve. When BM patients are introduced to treatment options, studies have found patients rate HRQOL among the most important factors when making treatment decisions [107]. Historically, clinical studies have focused on increasing survival outcomes; only in recent decades have studies incorporated endpoints for neurocognitive function and HRQOL. As clinical trials are initiating the inclusion of these measurements, there is a need to harmonize the instruments used across studies. We are now equipped with validated tools to measure cognitive function and HRQOL for patients with BMs and, therefore, should design later-phase trials to include a standardized battery of tests that have potential for adoption in both the academic and community setting.

## Figures and Tables

**Table 1:** Tools for measuring QOL in BM patients.

Screening Tools	Description	Number of Questions/Time to Complete
EQ-5D	Measures mobility, self-care, usual activities, pain/discomfort, and anxiety/depression [71].	5 questions, <5 min
FACT-G	Questionnaire measuring physical, social, emotional, and functional well-being [72].	33 questions, 10–15 min
FACT-Br	Composed of the FACT-G and brain cancer subscale.	50 questions, 15–20 min
EORTC QLQ	EORTC QLQ-C30 is used to measure cancer patients’ physical, psychological, and social functioning [73]. Other forms are available; EORTC QLQ-BN20 is designed for brain cancer patients.	30 questions, 10 min

**Table 2:** Tools for measuring cognitive function in BM patients.

Screening Tools	Description	Number of Questions/Time to Complete
MMSE	Brief screen measuring mental status, including orientation, language, memory, and other abilities [74].	30 questions, 10 min [94]
MoCA	Assesses short-term memory recall, visuospatial abilities (clock drawing and copying a 3-dimensional cube), executive function, attention, language, abstract reasoning, and orientation to time and place.	30 points, <10 min
RBANS	Tests immediate memory, visuospatial abilities, language, attention, and delayed memory.	12 subtests, 30 min
TMT	Assesses visual scanning, graphomotor speed, and executive function [95].	2 tests, 3–5 min
HVLT-R	Assesses verbal learning, immediate recall, delayed recall, and recognition [96]. Various forms available.	12 items for 3 learning trials, a free recall and recognition section, 10–15 min plus delayed recall time
COWAT	Measures phonemic verbal fluency [91].	3 letters, 3 min total
